# Crystal structure of di­ethyl­ammonium dioxido{*Z*)-*N*-[(pyri­din-2-yl)car­bon­yl­azan­idyl]pyri­dine-2-car­box­imid­ato}vana­date(1−) monohydrate

**DOI:** 10.1107/S2056989024001166

**Published:** 2024-02-08

**Authors:** Bipul Mondal

**Affiliations:** a Department of Chemistry, Ramakrishna Mission Vivekananda Centenary College, Rahara, Kolkata-700118, West Bengal, India; University of Missouri-Columbia, USA

**Keywords:** crystal structure, dioxidovanadium(V), *O*,*N*,*O* -donor Schiff-base, hydrogen bond

## Abstract

The title compound, (C_4_H_12_N)[V(C_12_H_8_N_4_O_2_)O_2_]·H_2_O, was synthesized *via* aerial oxidation on refluxing picolinohydrazide with ethyl picolinate followed by addition of V^IV^O(acac)_2_ and di­ethyl­amine in methanol. It crystallizes in the triclinic crystal system in space group *P*  . In the complex anion, the dioxidovanadium(V) moiety exhibits a distorted square-pyramidal geometry. In the crystal, extensive hydrogen bonding links the water mol­ecule to two complex anions and one di­ethyl­ammonium ion. One of the CH_2_ groups in the di­ethyl­amine is disordered over two sets of sites in a 0.7:0.3 ratio.

## Abstract 2

A new ionic dioxidovanadium(V) compound with an *O*,*N*,*O* donor ligand is reported. In the crystal, extensive hydrogen bonding is observed.

## 1. Chemical context

Vanadium, a biologically important trace element with the +V oxidation state, has received considerable attention among the three (*viz*., +III, +IV and +V) physiologically important oxidation states. In this oxidation state, vanadium exists in three different motifs, *viz*., VO^3+^, V_2_O_3_^4+^ and VO_2_^+^. The formation and stability of these three motifs depends upon the nature of the solvent, the pH of the reaction medium and basicity of the donor atoms of the ligand(s), with a preference for N, O-donor ligands because of the hard acidic nature of V^V^. It is evident from the literature (Mondal *et al.*, 2010 ▸, 2008 ▸) that the vanadium complexes containing VO_2_^+^ motifs are formed in basic media. Vanadium compounds show the catalytic cycle of haloperoxidase activity has been suggested to proceed through hydrogen-bonding inter­actions (Colpas *et al.*, 1996 ▸; Messerschimdt & Wever, 1996 ▸; Weyand *et al.*, 1999 ▸; Isupov *et al.*, 2000 ▸). In the presence of appropriate hydrogen-bond donors, hydrogen bonding is a general feature of vanadium(IV) and vanadium(V) complexes (Mondal *et al.*, 2010 ▸; Plass, 1997 ▸, 1998 ▸; Plass & Yozgatli, 2003 ▸; Pohlmann & Plass, 2001 ▸; Pohlmann *et al.*, 2005 ▸; Vergopoulos *et al.*, 1993 ▸; Sutradhar *et al.*, 2006 ▸). In general, these examples lead to the formation of hydrogen-bonded mol­ecular assemblies ranging from simple dimers to three-dimensional networks. 
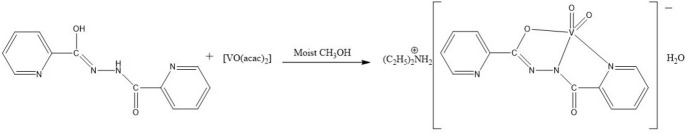


In this work, an ionic compound of dioxidovanadium(V) containing a symmetric *N*-(pyridine-2-ylcarbamo­yl)picolinamide (Shao *et al.*, 1999 ▸) ligand (H_2_ *L)* bound to vanadium through NNO in an asymmetric fashion, was synthesized in the presence of di­ethyl­amine in good yield and characterized by X-ray crystallography. The title compound may be used for anti­diabetic drug development (Jia *et al.*, 2017 ▸).

## 2. Structural commentary

**Figure 1:**
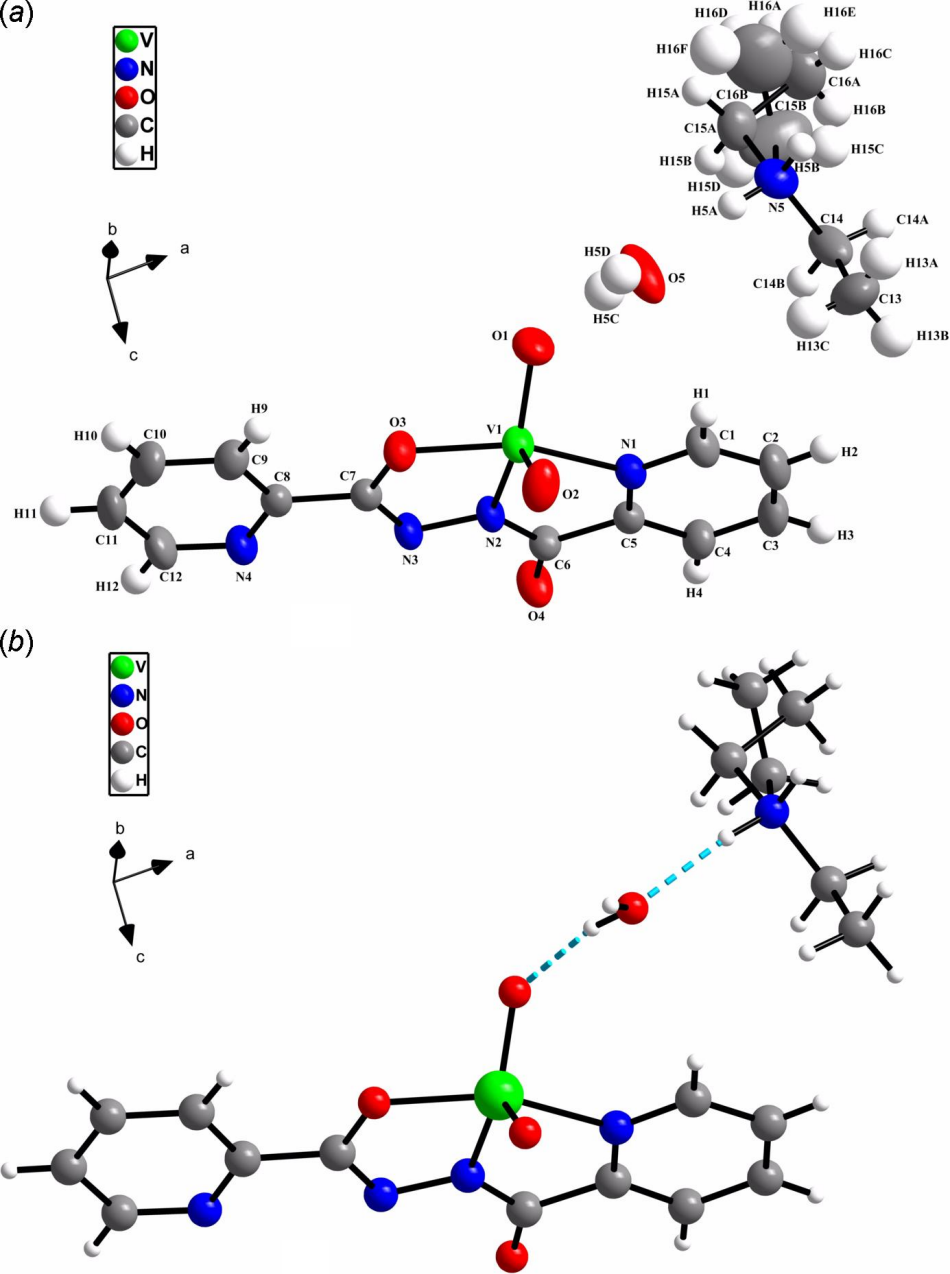
(*a*) The mol­ecular structure with the atom-numbering scheme and ellipsoids drawn at the 50% probability level and (*b*) the intra­molecular hydrogen bond.

**Figure 2:**
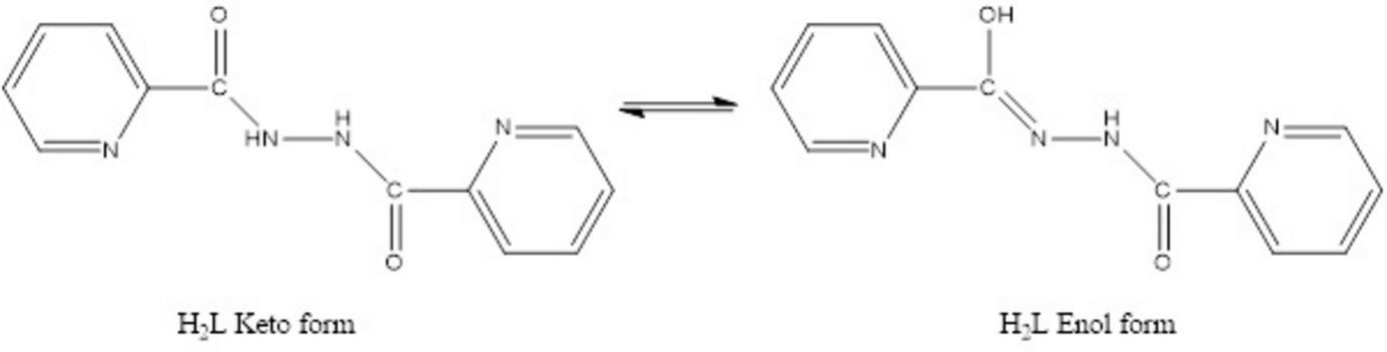
The keto–enol tautomeric forms of the ligand.

The solid-state mol­ecular structure was confirmed by single-crystal X-ray characterization. The title compound crystallizes in the triclinic crystal system, space group *P* 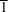 . The asymmetric unit (Fig. 1 ▸ *a*) comprises a di­ethyl­ammonium cation, a complex dioxidovanadium(V) and a water mol­ecule, which is inter­linked between the two ionic parts of the compound through hydrogen bonding (Fig. 1 ▸ *b*). The anionic part of the compound consists of one crystallographically independent V^5+^ ion, two oxido ligands and one NNO donor ligand with coordination sphere of the VO_3_N_2_ type (Fig. 1 ▸ *a*). The V^5+^ ion is coordinated by two oxygen (O1 and O2) atoms (oxido ligands), one nitro­gen (N1) atom of the pyridine ring, one deprotonated amide nitro­gen (N2) atom and a deprotonated amide-oxygen (O3) through enolization (Fig. 2 ▸) of the ligand. The five-coordinate V^5+^ ion has a distorted square-pyramidal geometry with one of the two oxido oxygen atoms (O1) at the apex. The extent of distortion from a perfect square-pyramidal geometry can be qu­anti­fied by the structural index parameter (τ = 0.35), as determined from the equation τ = (β - α)/60 (where β and α are the two largest *L*—*M*—*L* angles), which is 0 for an ideal­ized square pyramid and 1 for a trigonal bipyramid (Nair *et al.*, 2018 ▸; Ghosh *et al.*, 2022 ▸). The square plane consists of one nitro­gen atom from the pyridine ring (N1), one deprotonated amide nitro­gen (N2), one enolate oxygen (O3) and one oxido oxygen (O2) atom of the ligands. The vanadium atom is located 0.555 (4) Å above the equatorial plane and displaced towards the axial O1 atom. Selected bonds involving the V atom are given in Table 1 ▸. The V—O1 bond is longer than V—O2, probably due to the involvement of O1 in a hydrogen bond with the water hydrogen atom H5*C* (Table 2 ▸). Among the three V—O bonds, the longest is the V—O3 bond length due to the absence of a V—O π-bond (Mondal *et al.*, 2010 ▸; Jia *et al.*, 2017 ▸). In the absence of di­ethyl­amine, the formation of neutral dioxido complex has been reported in which the uncoordinated pyridine atom N4 is protonated (Jia *et al.*, 2017 ▸), but in this case the protonation of the di­ethyl­amine moiety (p*K*_a_ = 10.98) is probably due to its higher basicity than pyridine (p*K*_a_ = 5.23).

## 3. Supra­molecular features

**Figure 4:**
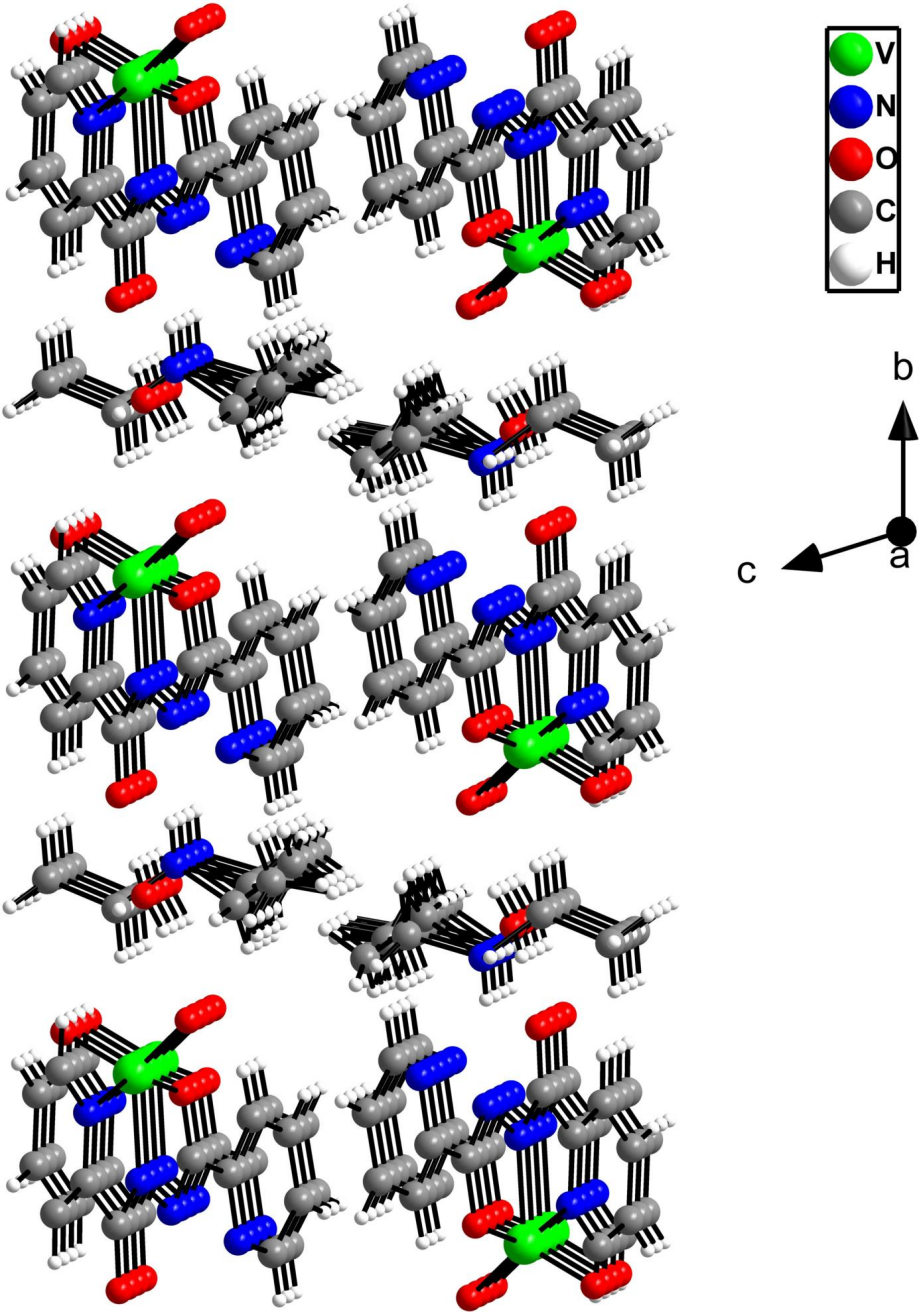
The three-dimensional packing arrangement of the components of the title compound.

The oxygen (O5) atom of water acts as a hydrogen-bond donor with an acceptor oxido group (O1) of the dioxidovan­adium(V) complex in the same asymmetric unit (O5—H5*C*⋯O1) and a symmetry-related amide oxygen (O4) atom in a neighbouring asymmetric unit (O5—H5*D*⋯O4) (Table 2 ▸). The O5 atom also acts as a hydrogen-bond acceptor for the amine H5*A* atom (N5—H5*A*⋯O5). Another amine hydrogen (H5*B*) is hydrogen bonded with the N3 atom (N5—H5*B*⋯N3) of an adjacent complex. Two C—H⋯O and one C—H⋯N inter­actions are also observed. These hydrogen bonds within the same and different asymmetric units enhance the crystal packing of the compounds (Figs. 1 ▸ *b* and 3 ▸). The mono-periodic constructs are packed perpendicular to the *bc* plane, giving rise to an overall three-dimensional packing arrangement (Fig. 4 ▸).

## 4. Database survey

Mondal *et al.* (2010 ▸) reported numerous ionic dioxidovanadium(V) compounds with *O*,*N*,*O* donor ligands in presence of different types of bases. Jia *et al.* (2017 ▸) also reported a neutral dioxidovanadium(V) compound with the same ligand.

## 5. Synthesis and crystallization

To a solution of picolinohydrazide (0.137 g, 1 mmol) in methanol (25 ml) was added ethyl picolinate (0.151 g, 1 mmol). The solution was heated under reflux for 3 h. The reaction mixture was cooled to room temperature and a methano­lic solution (20 ml) of [V^IV^O(acac)_2_] (0.265 g, 1 mmol) was added with stirring. After stirring for 2 h, a methano­lic solution (10 ml) of di­ethyl­amine (1 ml) was added with continuous stirring. The solution immediately turned yellow and the reaction mixture was then refluxed for 1 h. The reaction mixture was then kept for slow evaporation at room temperature. A yellow X-ray quality crystalline compound was obtained, which was filtered, washed with methanol and dried over silica gel (fused). Yield: 0.34 g (82%). Crystals of the complex were obtained after 4-days on slow evaporation at room temperature.

## 6. Refinement

Crystal data, data collection and structure refinement details are summarized in Table 3 ▸. N-bound H atoms were refined with *U_i_*_so_(H) = 1.2*U*_eq_(N). C-bound H atoms and water H atoms were placed at calculated positions (C—H = 0.93–0.97 Å, O—H = 0.85 Å) and refined as riding with *U*_iso_(H) = 1.2*U*_eq_(C) or 1.5*U*_eq_(C-methyl,O). Initially, residual electron density ws noted near to C15. The part command was used to locate the two positions of C15 (*i.e.*, C15*A*, in PART 1; and C15*B*, in PART 2). The site occupancie are 0.7 and 0.3, respectively. Subsequently, an isotropic refinement was done and finally, an anisotropic refinement is performed.

## Supplementary Material

Crystal structure: contains datablock(s) I. DOI: 10.1107/S2056989024001166/ev2002sup1.cif

Structure factors: contains datablock(s) I. DOI: 10.1107/S2056989024001166/ev2002Isup2.hkl

CCDC reference: 2294358

Additional supporting information:  crystallographic information; 3D view; checkCIF report

## Figures and Tables

**Figure 3:**
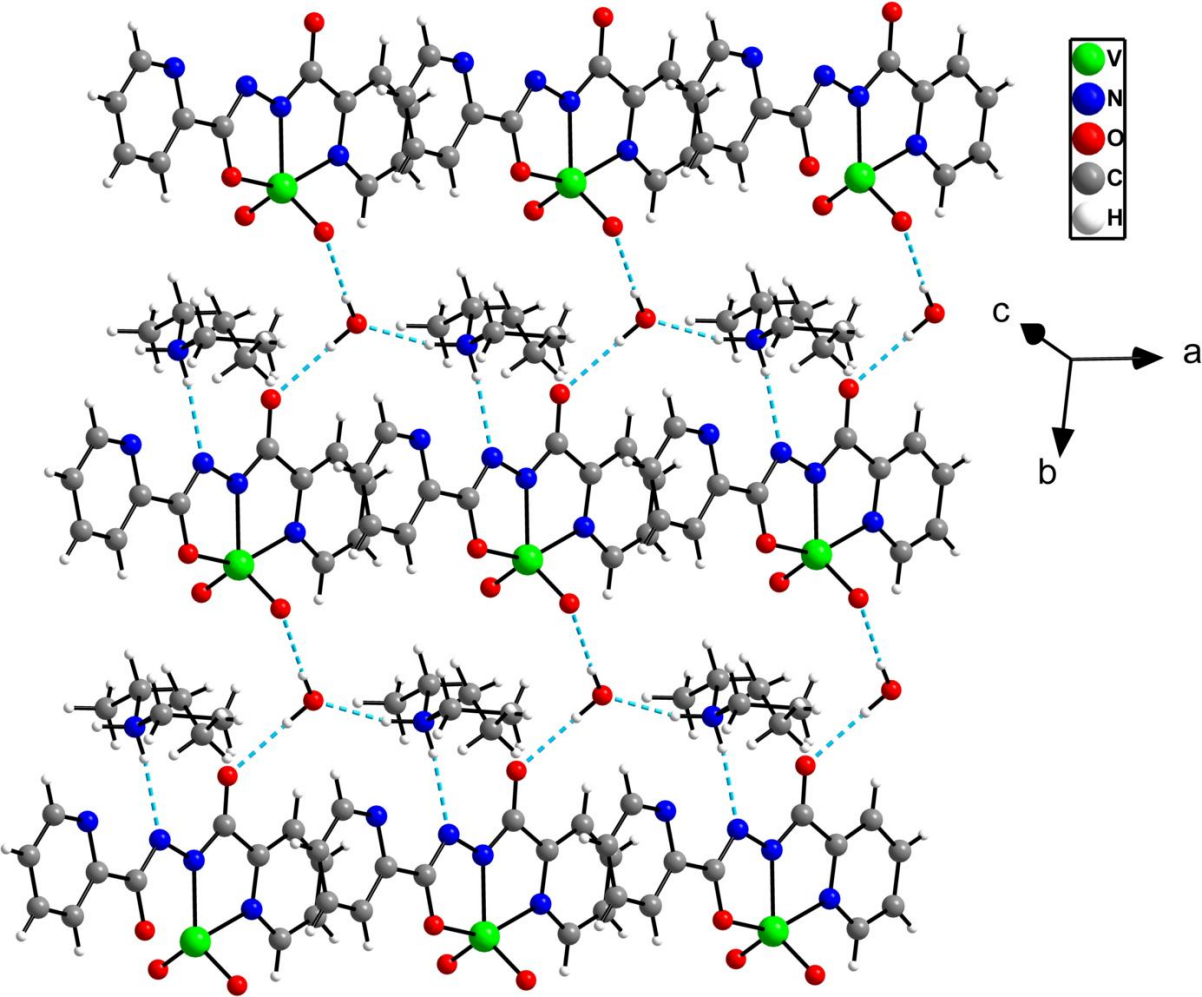
The hydrogen bonding in adjacent asymmetric units.

**Table 1:** Selected bond lengths (Å)

V1—O2	1.6107 (15)	V1—N2	2.0385 (15)
V1—O3	1.9461 (14)	V1—N1	2.1170 (15)
V1—O1	1.6310 (16)		

**Table 2:** Hydrogen-bond geometry (Å, °)

*D*—H⋯*A*	*D*—H	H⋯*A*	*D*⋯*A*	*D*—H⋯*A*
N5—H5*A*⋯O5	0.95 (3)	1.92 (3)	2.848 (3)	165 (3)
N5—H5*B*⋯N3^i^	0.85 (3)	2.08 (3)	2.918 (3)	171 (3)
O5—H5*C*⋯O1	0.85	1.94	2.776 (3)	169
O5—H5*D*⋯O4^ii^	0.85	1.99	2.838 (2)	178
C2—H2⋯O2^iii^	0.93	2.48	3.277 (3)	143
C14—H14*A*⋯O4^i^	0.97	2.57	3.172 (3)	121
C15*A*—H15*A*⋯N4^i^	0.97	2.59	3.233 (5)	124

Symmetry codes: (i) 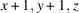 ; (ii) 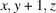 ; (iii) 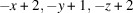 .

**Table 3:** Experimental details

Crystal data	
Chemical formula	(C_4_H_12_N)[V(C_12_H_8_N_4_O_2_)O_2_]·H_2_O
*M*_r_	415.32
Crystal system, space group	Triclinic, *P* 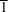
Temperature (K)	298
*a*, *b*, *c* (Å)	7.6850 (4), 9.4135 (4), 13.9147 (7)
α, β, γ (°)	105.609 (2), 101.103 (2), 96.253 (2)
*V* (Å^3^)	937.50 (8)
*Z*	2
Radiation type	Mo *K*α
μ (mm^−1^)	0.57
Crystal size (mm)	0.32 × 0.18 × 0.03

Data collection	
Diffractometer	Bruker D8 Quest with Photon II area detector
Absorption correction	Multi-scan (*SADABS*; Krause *et al.*, 2015 ▸)
*T*_min_, *T*_max_	0.670, 0.747
No. of measured, independent and observed [*I* > 2σ(*I*)] reflections	61285, 5048, 3852
*R*_int_	0.081
(sin θ/λ)_max_ (Å^−1^)	0.685

Refinement	
*R*[*F* ^2^ > 2σ(*F* ^2^)], *wR*(*F* ^2^), *S*	0.038, 0.110, 1.06
No. of reflections	5048
No. of parameters	274
H-atom treatment	H atoms treated by a mixture of independent and constrained refinement
Δρ_max_, Δρ_min_ (e Å^−3^)	0.43, −0.39

Computer programs: *APEX2* and *SAINT* (Bruker, 2016 ▸), *SHELXT2018/2* (Sheldrick, 2015*a* ▸), *SHELXL* (Sheldrick, 2015*b* ▸) and *OLEX2* (Dolomanov *et al.*, 2009 ▸).

## supplementary crystallographic information

### Crystal data

**Table:**

(C_4_H_12_N)[V(C_12_H_8_N_4_O_2_)O_2_]·H_2_O	*Z* = 2
*M**_r_* = 415.32	*F*(000) = 432
Triclinic, *P*1	*D*_x_ = 1.471 Mg m^−^^3^
*a* = 7.6850 (4) Å	Mo *K*α radiation, λ = 0.71073 Å
*b* = 9.4135 (4) Å	Cell parameters from 9990 reflections
*c* = 13.9147 (7) Å	θ = 2.3–30.2°
α = 105.609 (2)°	µ = 0.57 mm^−^^1^
β = 101.103 (2)°	*T* = 298 K
γ = 96.253 (2)°	BLOCK, yellow
*V* = 937.50 (8) Å^3^	0.32 × 0.18 × 0.03 mm

### Data collection

**Table:**

Bruker D8 Quest with Photon II area detector diffractometer	3852 reflections with *I* > 2σ(*I*)
φ and ω scans	*R*_int_ = 0.081
Absorption correction: multi-scan (SADABS; Krause *et al.*, 2015)	θ_max_ = 29.1°, θ_min_ = 2.3°
*T*_min_ = 0.670, *T*_max_ = 0.747	*h* = −10→10
61285 measured reflections	*k* = −12→12
5048 independent reflections	*l* = −19→19

### Refinement

**Table:**

Refinement on *F*^2^	0 restraints
Least-squares matrix: full	Hydrogen site location: mixed
*R*[*F*^2^ > 2σ(*F*^2^)] = 0.038	H atoms treated by a mixture of independent and constrained refinement
*wR*(*F*^2^) = 0.110	*w* = 1/[σ^2^(*F*_o_^2^) + (0.045*P*)^2^ + 0.4439*P*] where *P* = (*F*_o_^2^ + 2*F*_c_^2^)/3
*S* = 1.06	(Δ/σ)_max_ = 0.001
5048 reflections	Δρ_max_ = 0.43 e Å^−^^3^
274 parameters	Δρ_min_ = −0.39 e Å^−^^3^

### Special details

**Table:**

Geometry. All esds (except the esd in the dihedral angle between two l.s. planes) are estimated using the full covariance matrix. The cell esds are taken into account individually in the estimation of esds in distances, angles and torsion angles; correlations between esds in cell parameters are only used when they are defined by crystal symmetry. An approximate (isotropic) treatment of cell esds is used for estimating esds involving l.s. planes.

### Fractional atomic coordinates and isotropic or equivalent isotropic displacement parameters (Å^2^)

**Table:**

	*x*	*y*	*z*	*U*_iso_*/*U*_eq_	Occ. (<1)
V1	0.58085 (4)	0.29537 (3)	0.78356 (3)	0.03307 (10)	
N4	0.0021 (2)	−0.1100 (2)	0.63830 (14)	0.0431 (4)	
O2	0.5724 (2)	0.42364 (17)	0.88516 (12)	0.0518 (4)	
O3	0.32784 (18)	0.22542 (14)	0.71551 (11)	0.0394 (3)	
N3	0.36076 (19)	−0.00629 (16)	0.73136 (13)	0.0332 (3)	
C1	0.9836 (3)	0.3489 (2)	0.88556 (17)	0.0419 (5)	
H1	0.979921	0.446045	0.882458	0.050*	
O1	0.6564 (2)	0.36927 (18)	0.70301 (13)	0.0528 (4)	
C2	1.1439 (3)	0.3148 (3)	0.92898 (18)	0.0487 (5)	
H2	1.246761	0.387685	0.955215	0.058*	
O5	0.8727 (2)	0.6487 (2)	0.7639 (2)	0.0755 (6)	
H5C	0.799539	0.566441	0.738019	0.113*	
H5D	0.806183	0.714430	0.777364	0.113*	
N2	0.53498 (19)	0.07461 (16)	0.77552 (12)	0.0316 (3)	
C4	0.9945 (3)	0.0640 (2)	0.89249 (17)	0.0415 (4)	
H4	0.996177	−0.034329	0.893312	0.050*	
N5	1.2242 (3)	0.6901 (2)	0.72745 (16)	0.0484 (5)	
H5A	1.107 (4)	0.694 (3)	0.740 (2)	0.058*	
H5B	1.276 (4)	0.777 (3)	0.732 (2)	0.058*	
C3	1.1493 (3)	0.1707 (3)	0.93290 (18)	0.0493 (5)	
H3	1.256060	0.145211	0.962437	0.059*	
O4	0.64803 (19)	−0.13258 (15)	0.80366 (14)	0.0497 (4)	
N1	0.8323 (2)	0.24697 (17)	0.84751 (12)	0.0325 (3)	
C5	0.8380 (2)	0.1063 (2)	0.85105 (14)	0.0313 (4)	
C7	0.2627 (2)	0.08519 (19)	0.70259 (14)	0.0311 (4)	
C6	0.6610 (2)	0.00098 (19)	0.80739 (15)	0.0330 (4)	
C14	1.3191 (3)	0.6392 (3)	0.8113 (2)	0.0598 (7)	
H14A	1.442548	0.635076	0.805965	0.072*	
H14B	1.261482	0.539255	0.805202	0.072*	
C13	1.3170 (4)	0.7421 (4)	0.9123 (2)	0.0828 (10)	
H13A	1.371411	0.841452	0.917594	0.124*	
H13B	1.383288	0.709304	0.965860	0.124*	
H13C	1.194956	0.742057	0.918946	0.124*	
C15A	1.1933 (5)	0.5973 (5)	0.6175 (4)	0.0527 (10)	0.7
H15A	1.112883	0.639100	0.573984	0.063*	0.7
H15B	1.137207	0.496051	0.609451	0.063*	0.7
C16A	1.3721 (7)	0.5944 (7)	0.5852 (5)	0.0695 (13)	0.7
H16A	1.349650	0.557036	0.511873	0.104*	0.7
H16B	1.438875	0.530498	0.615382	0.104*	0.7
H16C	1.440214	0.693958	0.608190	0.104*	0.7
C8	0.0699 (2)	0.0330 (2)	0.65164 (14)	0.0326 (4)	
C9	−0.0329 (3)	0.1301 (2)	0.61863 (16)	0.0421 (5)	
H9	0.017119	0.229460	0.630326	0.050*	
C10	−0.2108 (3)	0.0772 (3)	0.56808 (19)	0.0534 (6)	
H10	−0.282243	0.140321	0.544890	0.064*	
C11	−0.2801 (3)	−0.0687 (3)	0.55262 (19)	0.0554 (6)	
H11	−0.398965	−0.107782	0.517707	0.067*	
C12	−0.1699 (3)	−0.1567 (3)	0.58996 (19)	0.0526 (6)	
H12	−0.219043	−0.255526	0.580788	0.063*	
C15B	1.279 (3)	0.5710 (13)	0.6404 (12)	0.102 (6)	0.3
H15C	1.399256	0.553432	0.665826	0.123*	0.3
H15D	1.196845	0.477106	0.621828	0.123*	0.3
C16B	1.276 (3)	0.6182 (19)	0.5559 (13)	0.116 (6)	0.3
H16D	1.270778	0.533969	0.497547	0.173*	0.3
H16E	1.383543	0.688974	0.567037	0.173*	0.3
H16F	1.172786	0.664919	0.543386	0.173*	0.3

### Atomic displacement parameters (Å^2^)

**Table:**

	*U*^11^	*U*^22^	*U*^33^	*U*^12^	*U*^13^	*U*^23^
V1	0.03157 (16)	0.02325 (15)	0.04138 (19)	0.00202 (11)	0.00358 (13)	0.00908 (12)
N4	0.0319 (8)	0.0415 (9)	0.0525 (11)	−0.0027 (7)	0.0019 (7)	0.0175 (8)
O2	0.0457 (8)	0.0400 (8)	0.0543 (9)	0.0124 (7)	−0.0010 (7)	−0.0046 (7)
O3	0.0345 (7)	0.0278 (6)	0.0520 (8)	0.0024 (5)	−0.0009 (6)	0.0141 (6)
N3	0.0249 (7)	0.0277 (7)	0.0438 (9)	−0.0003 (6)	0.0023 (6)	0.0111 (6)
C1	0.0354 (10)	0.0326 (9)	0.0495 (12)	−0.0043 (8)	0.0055 (9)	0.0056 (9)
O1	0.0511 (9)	0.0474 (9)	0.0672 (11)	0.0019 (7)	0.0118 (8)	0.0329 (8)
C2	0.0319 (10)	0.0489 (12)	0.0526 (13)	−0.0062 (9)	0.0018 (9)	0.0049 (10)
O5	0.0432 (9)	0.0467 (10)	0.146 (2)	0.0060 (8)	0.0276 (11)	0.0400 (12)
N2	0.0239 (7)	0.0261 (7)	0.0422 (9)	0.0002 (5)	0.0031 (6)	0.0106 (6)
C4	0.0325 (9)	0.0428 (11)	0.0485 (12)	0.0072 (8)	0.0035 (8)	0.0163 (9)
N5	0.0555 (12)	0.0325 (9)	0.0583 (12)	0.0029 (8)	0.0163 (10)	0.0151 (9)
C3	0.0294 (10)	0.0594 (14)	0.0536 (13)	0.0053 (9)	−0.0009 (9)	0.0162 (11)
O4	0.0362 (7)	0.0312 (7)	0.0835 (12)	0.0050 (6)	0.0057 (7)	0.0260 (7)
N1	0.0288 (7)	0.0288 (7)	0.0371 (8)	0.0010 (6)	0.0065 (6)	0.0076 (6)
C5	0.0283 (8)	0.0318 (9)	0.0339 (9)	0.0043 (7)	0.0067 (7)	0.0108 (7)
C7	0.0306 (8)	0.0289 (8)	0.0324 (9)	0.0018 (7)	0.0058 (7)	0.0090 (7)
C6	0.0320 (9)	0.0262 (8)	0.0399 (10)	0.0035 (7)	0.0040 (7)	0.0119 (7)
C14	0.0450 (12)	0.0587 (15)	0.098 (2)	0.0169 (11)	0.0232 (13)	0.0522 (15)
C13	0.072 (2)	0.124 (3)	0.0664 (19)	0.0195 (19)	0.0103 (15)	0.054 (2)
C15A	0.049 (2)	0.041 (2)	0.059 (3)	0.0063 (15)	0.0060 (18)	0.0045 (17)
C16A	0.066 (3)	0.077 (3)	0.065 (3)	0.019 (2)	0.023 (2)	0.011 (2)
C8	0.0296 (8)	0.0355 (9)	0.0317 (9)	0.0034 (7)	0.0066 (7)	0.0095 (7)
C9	0.0372 (10)	0.0438 (11)	0.0438 (11)	0.0103 (8)	0.0046 (8)	0.0127 (9)
C10	0.0353 (11)	0.0700 (16)	0.0564 (14)	0.0171 (11)	0.0032 (10)	0.0232 (12)
C11	0.0260 (9)	0.0803 (17)	0.0554 (14)	0.0011 (10)	0.0004 (9)	0.0221 (13)
C12	0.0344 (11)	0.0547 (13)	0.0609 (15)	−0.0099 (9)	0.0015 (10)	0.0178 (11)
C15B	0.179 (19)	0.039 (5)	0.083 (10)	0.028 (9)	0.019 (12)	0.013 (6)
C16B	0.137 (17)	0.088 (10)	0.111 (14)	0.029 (11)	0.046 (13)	−0.005 (9)

### Geometric parameters (Å, º)

**Table:**

V1—O2	1.6107 (15)	C4—C5	1.378 (3)
V1—O3	1.9461 (14)	N5—C14	1.473 (3)
V1—O1	1.6310 (16)	N5—C15A	1.502 (5)
V1—N2	2.0385 (15)	N5—C15B	1.573 (16)
V1—N1	2.1170 (15)	O4—C6	1.237 (2)
N4—C8	1.340 (2)	N1—C5	1.343 (2)
N4—C12	1.329 (3)	C5—C6	1.506 (2)
O3—C7	1.311 (2)	C7—C8	1.480 (2)
N3—N2	1.400 (2)	C14—C13	1.481 (4)
N3—C7	1.296 (2)	C15A—C16A	1.526 (7)
C1—C2	1.375 (3)	C8—C9	1.383 (3)
C1—N1	1.342 (2)	C9—C10	1.380 (3)
C2—C3	1.377 (3)	C10—C11	1.362 (4)
N2—C6	1.327 (2)	C11—C12	1.374 (3)
C4—C3	1.385 (3)	C15B—C16B	1.36 (2)
			
O2—V1—O3	102.55 (7)	C1—N1—V1	123.69 (13)
O2—V1—O1	110.64 (9)	C1—N1—C5	118.88 (16)
O2—V1—N2	121.01 (8)	C5—N1—V1	117.43 (12)
O2—V1—N1	95.14 (7)	C4—C5—C6	123.33 (17)
O3—V1—N2	74.82 (5)	N1—C5—C4	121.92 (17)
O3—V1—N1	148.99 (6)	N1—C5—C6	114.75 (15)
O1—V1—O3	102.22 (7)	O3—C7—C8	117.03 (15)
O1—V1—N2	127.77 (8)	N3—C7—O3	122.55 (16)
O1—V1—N1	94.92 (7)	N3—C7—C8	120.41 (16)
N2—V1—N1	74.24 (6)	N2—C6—C5	109.38 (15)
C12—N4—C8	116.92 (18)	O4—C6—N2	129.15 (17)
C7—O3—V1	117.29 (11)	O4—C6—C5	121.47 (16)
C7—N3—N2	106.98 (14)	N5—C14—C13	110.7 (2)
N1—C1—C2	122.3 (2)	N5—C15A—C16A	109.9 (3)
C1—C2—C3	118.8 (2)	N4—C8—C7	117.44 (16)
N3—N2—V1	118.33 (10)	N4—C8—C9	122.41 (18)
C6—N2—V1	124.19 (12)	C9—C8—C7	120.14 (17)
C6—N2—N3	117.46 (14)	C10—C9—C8	119.0 (2)
C5—C4—C3	118.71 (19)	C11—C10—C9	119.1 (2)
C14—N5—C15A	120.9 (2)	C10—C11—C12	118.2 (2)
C14—N5—C15B	94.6 (6)	N4—C12—C11	124.4 (2)
C2—C3—C4	119.43 (19)	C16B—C15B—N5	111.2 (11)

### Hydrogen-bond geometry (Å, º)

**Table:**

*D*—H···*A*	*D*—H	H···*A*	*D*···*A*	*D*—H···*A*
N5—H5*A*···O5	0.95 (3)	1.92 (3)	2.848 (3)	165 (3)
N5—H5*B*···N3^i^	0.85 (3)	2.08 (3)	2.918 (3)	171 (3)
O5—H5*C*···O1	0.85	1.94	2.776 (3)	169
O5—H5*D*···O4^ii^	0.85	1.99	2.838 (2)	178
C2—H2···O2^iii^	0.93	2.48	3.277 (3)	143
C14—H14*A*···O4^i^	0.97	2.57	3.172 (3)	121
C15*A*—H15*A*···N4^i^	0.97	2.59	3.233 (5)	124

Symmetry codes: (i) *x*+1, *y*+1, *z*; (ii) *x*, *y*+1, *z*; (iii) −*x*+2, −*y*+1, −*z*+2.
